# Investigation on Water Transformation and Pore Structure of Cement-Stabilized Dredged Sediment Based on NMR Technology

**DOI:** 10.3390/ma15093178

**Published:** 2022-04-28

**Authors:** Shiquan Wang, Xingxing He, Guanghua Cai, Lei Lang, Hongrui Ma, Shunmei Gong, Zhiyong Niu

**Affiliations:** 1 National Engineering Research Center of Coal Mine Water Hazard Controlling, School of Resources and Civil Engineering, Suzhou University, Suzhou 234000, China; wsq@ahszu.edu.cn (S.W.); wsq_cersm@163.com (H.M.); hxx_cersm@163.com (S.G.); nzy1232022@163.com (Z.N.); 2 State Key Laboratory of Hydroscience and Engineering, Tsinghua University, Beijing 100084, China; 3 School of Civil Engineering, Nanjing Forestry University, Nanjing 210037, China; 4 State Key Laboratory of Geomechanics and Geotechnical Engineering, Institute of Rock and Soil Mechanics, Chinese Academy of Sciences, Wuhan 430071, China; budameiqian@163.com

**Keywords:** dredged sediment, cement-stabilized, NMR, permeability, strength, pore structure, water transformation

## Abstract

Cement-stabilized dredged sediment (CDS) when used as a new road construction material cannot only solve the problem of abandoned sediment disposal, but also effectively save natural soil resources. This study aimed to evaluate the strength and permeability of CDS and establish corresponding prediction models from the perspective of a stabilization mechanism. The soil–water composition and pore size distribution were investigated by the nuclear magnetic resonance (NMR) technique. The results demonstrated that more liquid pore water inside the CDS specimen transformed into combined water with cement hydration. The amount of combined water, which essentially characterized the hydration process of cement, presented a linear relationship with log (*t*). The cementation and filling action of hydrates resulted in the transformation of large pores into smaller ones, hence the optimal pore size decreased with an increasing curing period and cement content. The stress–strain curves and hydraulic conductivity were determined based on unconfined compression and flexible wall penetration tests, respectively. The unconfined compressive strength increased exponentially with the amount of combined water, and the functional correlations of hydraulic conductivity and micropore parameters were established. The reliability of the NMR technique as a new method to study the microscopic evolution mechanism of the strength and permeability of CDS was further verified by scanning electron microscopy and mercury intrusion porosimetry tests.

## 1. Introduction

Large quantities of sediments are dredged annually from harbors, waterways, and lakes to build port facilities, maintain shipping capacity, or protect water environment ecology [1,2,3]. The dredged sediments are considered waste due to their high-water content, high compressibility and low bearing capacity, and are difficult to be directly utilized without any treatment [4,5,6]. Cement-stabilized dredged sediment (CDS) when used as road construction material cannot only solve the problem of abandoned sediment disposal, but also effectively save natural soil resources [7,8,9,10]. Achour et al. [11] designed the method of road construction using solidified sludge and analyzed the road deflection and the tensile strength of borehole samples. Wang et al. [12] studied the effect of high curing temperature on the engineering performance of lime/cement-treated marine silt as an alternative material for road construction. Dubois et al. [13] developed a road construction material based on fine dredged sediments in combination with sand and binders, and the designed mixture met the threshold specified in the leaching toxicity test. The geomechanical properties of CDS have been extensively studied based on the cement content or soil–water/cement ratio in the past [14,15,16,17]. However, there are few studies that evaluate the strength and permeability of CDS and establish corresponding prediction models from the perspective of a stabilization mechanism.

Cement stabilization of dredged sediment involves following three aspects [18,19,20]: (1) the direct hydration reaction between cement and pore water in dredged sediment; (2) the secondary pozzolanic reaction between calcium hydroxide (cement hydration product) and active silicon aluminum dissolved from the clay minerals; and (3) the interaction between the soil aggregates and formed hydrates. When the cement is mixed into the dredged sediment, a large number of hydrates such as ettringite (AFt), calcium aluminate hydrate (CAH), calcium silicate hydrate (CSH), and calcium hydroxide (CH) are produced to bond the soil aggregates and fill the pores. Consequently, the internal structure of CDS becomes denser and firmer, resulting in an increase in strength and a decrease in hydraulic conductivity. Thus, it can be seen that the geomechanical properties of CDS are governed primarily by the number of hydrates. At the same time, the water in the CDS can be divided into liquid pore water (PW) and combined water (CW). The PW can be converted into the CW with cement hydration, resulting in the redistribution of water inside the CDS. Furthermore, the CW is an integral part of hydrates that can indirectly characterize the number of hydrates generated inside the CDS. Therefore, the real-time monitoring of the moisture state inside CDS can track the hydration reaction progress and reveal the evolution mechanism of the geomechanical properties (strength and permeability).

Recently, nuclear magnetic resonance (NMR) technology has been applied to detect water distribution and pore structure characteristics in the field of geotechnical engineering. Yu et al. [21] confirmed that the macrodynamic characteristics of soil observed in triaxial tests are closely related to the micro-pore size of soil obtained in the NMR test. Tian et al. [22] studied freezing–thawing characteristics of three soils by NMR technology and calculated the threshold and content of bound water in the soils. Yao et al. [23] investigated the pore–water status (including adsorbed water and capillary water) of expansive clay with different dry densities under the action of temperature and salt solution based on the NMR technique. NMR is a technique to investigate the content and distribution of protons (i.e., hydrogen nuclei) in a unit volume. Under the action of a magnetic field, the macroscopic magnetization vector of the proton group will be biased and out of balance. When the radio frequency stops, the proton group will recover from the non-equilibrium state to the equilibrium state. In this process, the nuclear magnetic signal starts to decay freely, which is referred to as the free induction decay (FID) curve. The peak point on the FID curve is proportional to the number of protons in the sample, so it can be used to determine the water content. At the same time, the shape of the FID curve is related to the transverse relaxation time (*T*_2_) of the proton, and the *T*_2_ distribution curve of pore water in the samples can be obtained by Fourier transformation [24]. In a uniform magnetic field, the *T*_2_ of liquid water was calculated as Equation (1),

(1) $$\frac{1}{T_{2}}=\rho_{2}{\left(\frac{S}{V}\right)}_{\mathrm{pore}}$$

where *ρ*_2_ is the transverse relaxation rate, a parameter characterizing the property of porous material, and *S* and *V* represent the surface area and volume of the pores where liquid water resided, respectively. When considering the pore as a cylindrical shape, Equation (1) can also be abbreviated as,

(2) $$\frac{1}{T_{2}}=\rho_{2}\frac{2}{R}$$

where *R* was the pore radius. Equations (1) and (2) show that the *T*_2_ value is proportional to the pore radius *R*, that is, a higher value of *T*_2_ represents a larger pore size [25]. Therefore, the *T*_2_ distribution curve can reflect the distribution of pore water and pore size in the porous media, and the peak area below the curve represents the corresponding water content [26].

Strength and permeability are the key performance parameters to evaluate the use of CDS as road building materials. This study aims to investigate the mechanism of water transformation and pore structure evolution during cement stabilization by the NMR technique and to establish a quantitative relationship between macro-geomechanical properties and microstructure. A series of laboratory specimens were prepared for different cement contents and curing periods. Furthermore, the stress–strain curves were determined based on an unconfined compression test, and the effect of the water content parameters on the strength development was quantitatively evaluated from the perspective of the stabilization mechanism. Similarly, mathematical models for evaluating the relationship between hydraulic conductivity, which are measured by a flexible wall permeameter and the representative parameters for pore size distribution, were proposed. Finally, the reliability of the NMR technique as a new method to study the microscopic evolution mechanism of strength and permeability of CDS was further verified by scanning electron microscopy (SEM) and mercury intrusion porosimetry (MIP) tests.

## 2. Materials and Methods

### 2.1. Raw Materials

**Figure 1:**
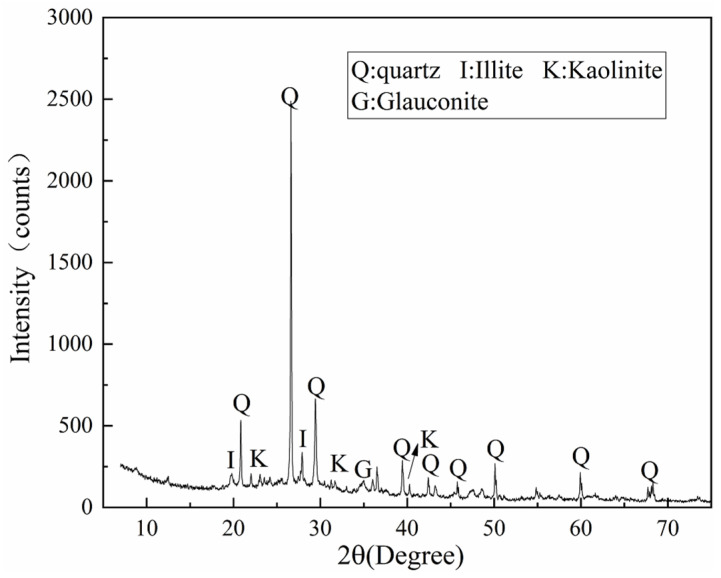
The XRD pattern of dredged sediment.

The dredged sediment used in this study was taken from the bottom of a river in Suzhou, China. The sediment collected from the site was first sieved to remove impurities with a large particle size. The basic physical properties of dredged sediment are shown in Table 1. According to the X-ray diffraction (XRD) test (Figure 1), the sediment was mainly composed of quartz, illite, kaolinite, and montmorillonite. According to the Unified Soil Classification System, the dredged sediment is classified as clay of high plasticity (CH). Ordinary Portland cement (P.O. 42.5) was used as the curing agent in this study, and the physical properties and chemical compositions are listed in Table 2.

### 2.2. Testing Methods

#### 2.2.1. NMR Test

**Figure 2:**
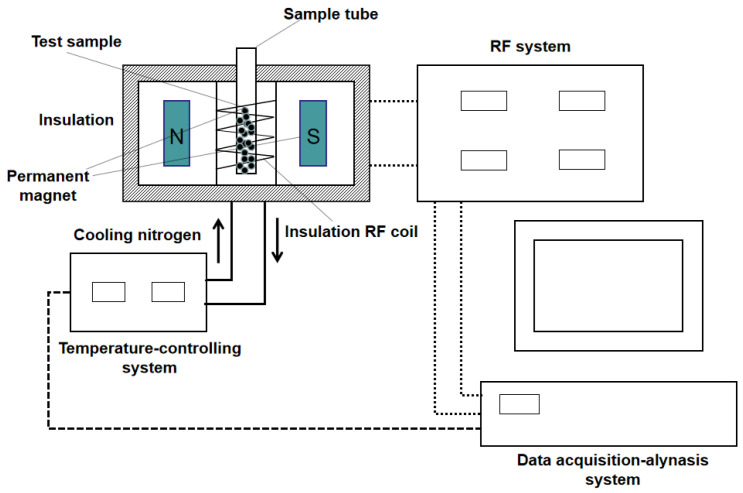
The structural principles of the NMR setup.

The FID curves of the CDS specimen were determined using the Carr-Purcell-Meiboom-Gill technique with a 23 MHz NMR setup [24]. The schematic diagram of the NMR experimental system is shown in Figure 2. The NMR equipment consists of a sample tube (Φ60 mm × H60 mm), data acquisition–analysis system, radio frequency (RF) system, and magnet unit. At the predetermined curing period, the sealed CDS specimens were put into the tube for NMR detection. Each NMR test can be completed in less than three minutes. After finishing all the detections, these sealed CDS specimens were put back into the cabinet for further curing. Then, the FID data were inverted and the *T*_2_ distribution curves were drawn. Based on the NMR theory: (1) the integral area under the *T*_2_ distribution curve (termed as the “peak area”) represents the total population of pore water molecules in the *T*_2_ range; (2) the horizontal coordinate of the curve (transverse relaxation time *T*_2_) represents the size of interstitial pores saturated with water; and (3) the “*T*_2_ at peak (*T*_2*P*_)” refers to the *T*_2_ value corresponding to the maximum NMR signal, thereby representing the optimal aperture [23].

#### 2.2.2. Geomechanical Tests

The unconfined compression test (UCT) was carried out by a hydraulic servo testing machine at a vertical loading rate of 1 mm/min, which was terminated when the peak strength of the specimen was attained or 8% of its axial strain was reached. The average value of three specimens was taken as the final unconfined compressive strength (UCS). The hydraulic conductivity testing (HCT) was carried out with the flexible wall permeameter. The specimen was vacuumized for 30 min and then saturated with deionized water for 24 h before the test. During the test, the confining pressure was kept at 200 kPa, and the osmotic pressure difference was kept at 100 kPa between the upper and lower ends of the specimens.

#### 2.2.3. Microscopic Experiment

The micromorphology of CDS was observed by using scanning electron microscopy (SEM, ZEISS MERLIN Compact, Muenchen, Germany). The pore structure was characterized by performing a mercury intrusion porosimetry (MIP) test, which can achieve a maximum pressure of 227 MPa. Sample pieces for microscopic analysis were immediately pre-treated with liquid nitrogen freezing and vacuum drying for 48 h. Furthermore, the blocks used for MIP and SEM analysis were taken from the undisturbed parallel CDS specimen to exclude the influence of micro-damage caused by external factors.

### 2.3. Mixed Design and Specimen Preparation

Table 3 shows the mixed design and corresponding test items. Based on the mass ratios of dry sediment, three different cement contents (5%, 10%, 15%) were added. Firstly, the retrieved sediment was prepared into a uniform slurry with an initial water content of 60% (1.2 times the liquid limit). Then, the prepared sediment paste was homogeneously mixed with a predetermined amount of cement inside a mechanical mixer as fast as possible. Afterward, the freshly stabilized CDS slurry was subsequently poured into cylindrical PVC molds (50 mm in diameter and 50 mm in height) and vibrated to dissipate the bubbles. The upper and lower ends of the mold were covered with plastic film to prevent water evaporation. The prepared specimens, together with the molds, were then transferred into a standard curing room (20 ± 2 °C, relative humidity ≥95%). Finally, the demolded specimens were sealed with a plastic membrane and cured until the predetermined period (i.e., 1, 3, 7, 14, 28 days). Six parallel specimens were prepared for each mixture.

## 3. Results and Discussion

### 3.1. NMR Test Results

#### 3.1.1. Characteristics of the *T*_2_ Distribution Curve

**Figure 3:**
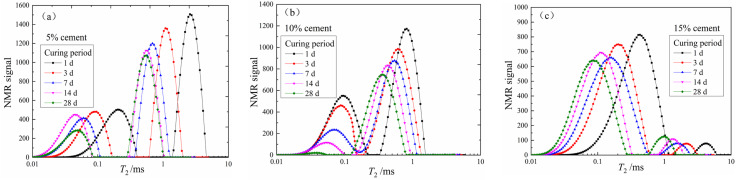
The *T*_2_ distribution curves of three groups of specimens: (**a**) 5% cement content, (**b**) 10% cement content, (**c**) 15% cement content.

The *T*_2_ distribution curves of three groups of specimens with different cement contents during the curing period were depicted in Figure 3a–c. It can be seen that the *T*_2_ distribution curves of all the specimens showed a bimodal distribution (primary peak and secondary peak) and spanned several orders of magnitude (0.01–10 ms). Since the pore size is proportional to relaxation time *T*_2_, the bimodal distributions indicated the existence of macro- and micro-pores within the CDS matrix. The *T*_2_ curves of the three groups of CDS specimens all shifted to the lower left with an increasing curing period. That is, the total peak area, the *T*_2_ at peak (*T*_2*P*_), and the maximum NMR signal gradually decreased, which denoted that the liquid PW inside the CDS specimen transformed into CW with cement hydration. At the same time, the proportion of large pores decreased and that of the small pores increased, and the distribution of internal pores became relatively uniform.

**Figure 4:**
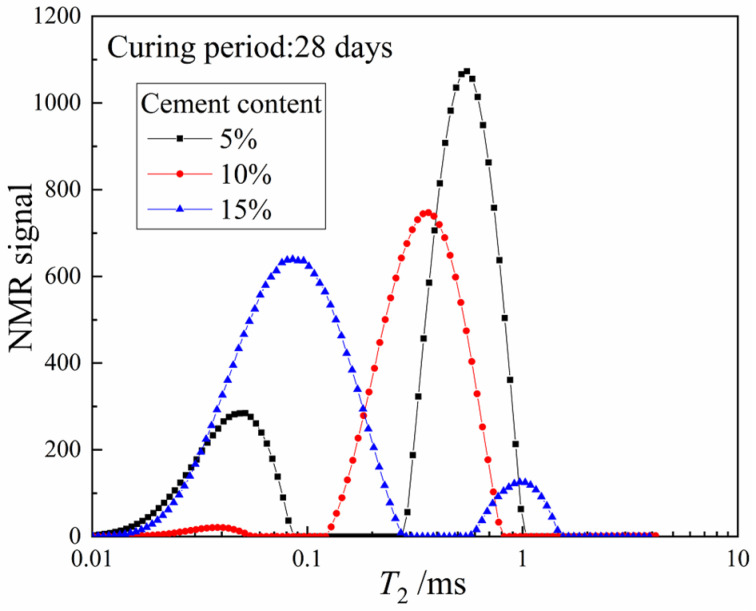
The 28-day *T*_2_ distribution curves of specimens with different cement content.

To highlight the influence of different cement contents more clearly, the *T*_2_ distribution curves of three groups of CDS specimens at the 28-day curing period are plotted in Figure 4. Similarly, the total peak area and the *T*_2*P*_ decreased gradually with increasing cement content. However, the main peak of the *T*_2_ distribution curves was on the left for the specimen with 5% and 10% cement, while that of the specimen with 15% cement was opposite, implying that the internal pore structure of CDS specimens changed significantly. Therefore, with the increase of cement content, more liquid PW was involved in cement hydration and transformed into CW, and the optimal pore size decreased while the proportion of small pores increased.

#### 3.1.2. Water Transformation Mechanism during Cement Stabilization

Since the CDS specimens were in sealed condition, the reduced liquid PW mass was exactly equal to the mass of generated CW. Given this, the corresponding water parameters were calculated as follows:

(I) The untreated dredged sediment with a known initial water content was taken as the standard sample, and its PW mass (*m*_pws_) and the corresponding total peak area of the *T*_2_ distribution curve (*A*_s_) were taken as the reference. Then, the PW mass corresponding to the unit peak area (*u*_pw_) was calculated:

(3) $$u_{\mathrm{pw}}=\frac{m_{\mathrm{pws}}}{A_{s}}$$

(II) The *T*_2_ distribution curve of each CDS specimen (shown in Figure 3) was integrated to calculate the peak area (*A*_i_), then the PW mass of each CDS (*m*_pwi_) was calculated:

(4) $$m_{\mathrm{pwi}}=u_{\mathrm{pw}}\times A_{i}=\frac{m_{\mathrm{pws}}}{A_{s}}\times A_{i}$$

(III) The difference between the initial PW mass (*m*_pw0_) and the PW mass (*m*_pwi_) of each CDS specimen was the CW mass generated by the hydration reaction (*m*_cw_):

(5) $$m_{\mathrm{cw}}=m_{\mathrm{pw}0}-m_{\mathrm{pwi}}$$

**Figure 5:**
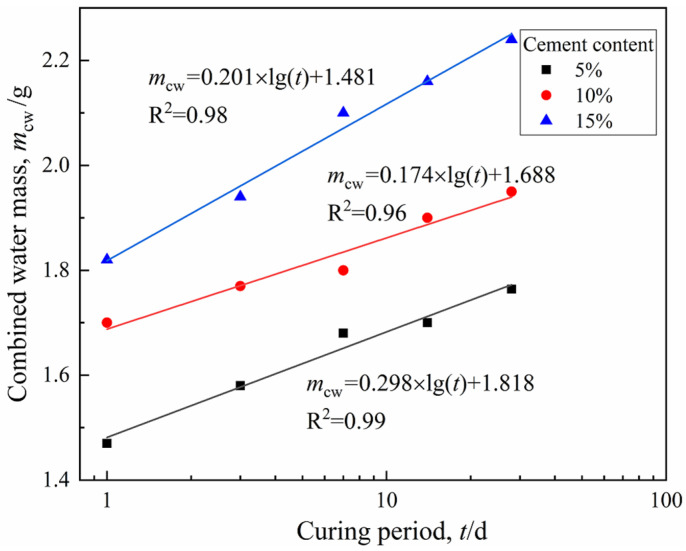
Relationship between CW mass and curing period.

**Figure 6:**
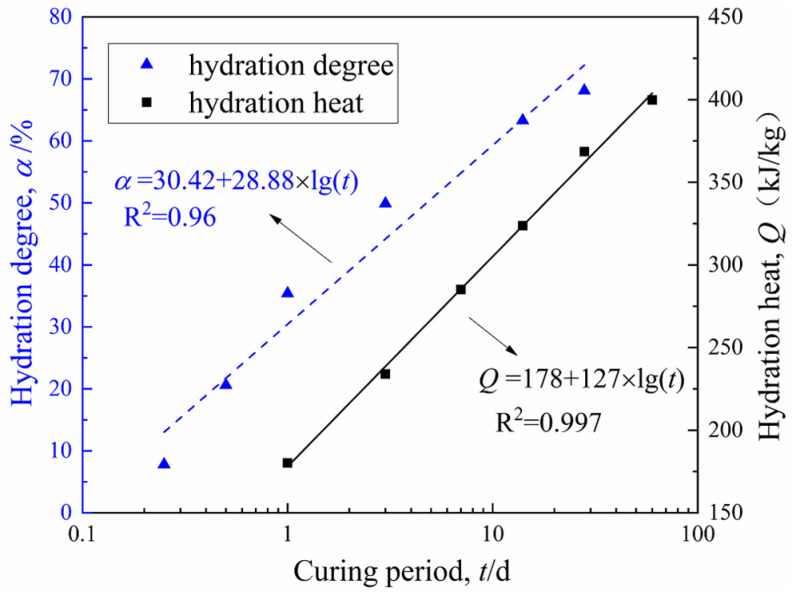
Cement hydration model established by Wang [27] and Dong [28].

**Figure 7:**
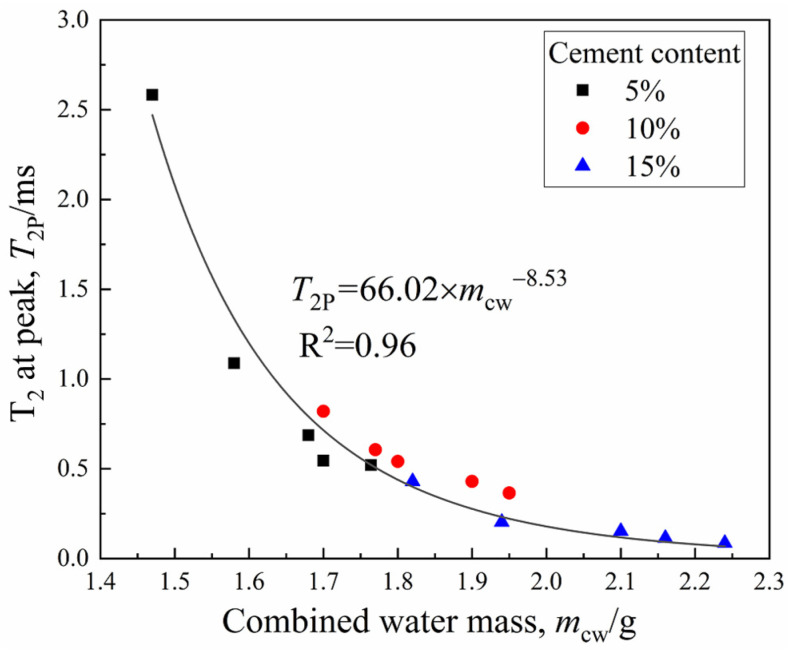
The *T*_2_ at peak versus the combined water.

Figure 5 shows the functional relationship between the CW mass and curing period of three groups of specimens. Figure 6 depicts the cement hydration model established by Wang and Dong, respectively [27,28], where hydration degree α = *Q*_(t)_/*Q*_(__max)_, *Q*_(t)_ was the hydration heat at time t, and *Q*_(__max)_ was the complete hydration heat. The model in this study indirectly represented the hydrates production in the CDS specimen from the perspective of water transformation, while Figure 6 shows the test results of the cement hydration heat measured by the heat of solution method. It is worth noting that *m*_cw_, α, or *Q*_(t)_ were all essential parameters that characterized cement hydration progress and all the presented linear relationships with log (*t*), which effectively proved the reliability of NMR technology in exploring the mechanism of water transformation in CDS. Moreover, it can be found by fitting the data in Figure 3 and Figure 5 that the variation of *T*_2*P*_ with *m*_cw_ also follows a power function form of y = a*x*^b^, which indicated the optimal pore size decreased with cement hydration, shown in Figure 7.

#### 3.1.3. Pore Structure Characteristics

**Figure 8:**
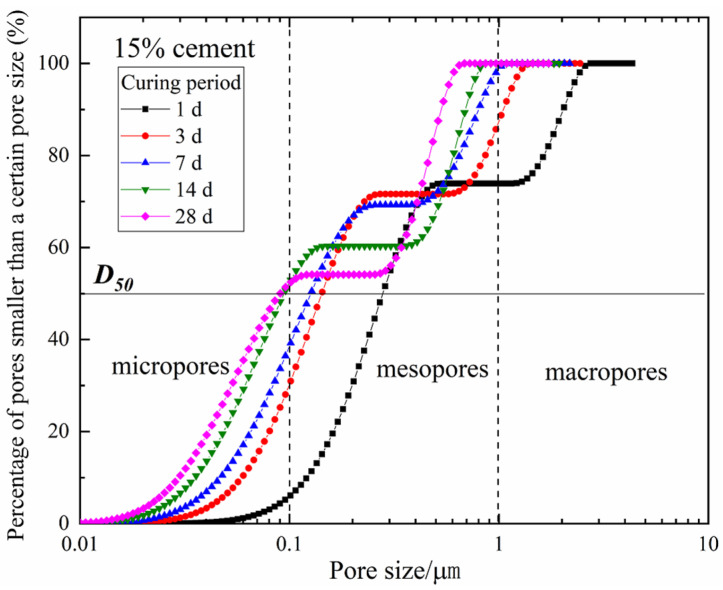
The cumulative pore size distribution curve of the specimen with 15% cement.

Combined with the research results of Tian et al. [24], the transverse relaxation rate, *ρ*_2_, of CDS is chosen as 0.22. Then, the cumulative pore size distribution curve can be obtained by calculating the *T*_2_ distribution data in Figure 3 with Equation (2). Taking the specimen with 15% cement as an example, the calculation results were plotted in Figure 8 (the ordinate indicates the percentage of pores smaller than a certain pore size). *D*_50_ is the median entrance throat diameter when the cumulative total pores reach 50%, which can be selected as a representative indicator to characterize the pore structure characteristics of the CDS matrix [29]. The *D*_50_ of specimens with 15% cement was 0.29, 0.14, 0.13, 0.10, and 0.09 µm for 1, 3, 7, 14, and 28 days, respectively, thereby indicating that the average pore size gradually decreased with the curing period. Furthermore, based on the feature and shape of the pore size distribution curve (Figure 8), the pore can be divided into three different grades by the pore diameter, namely “micropores” (<0.1 µm), “mesopores” (0.1~1.0 µm), and “macropores” (>1.0 µm) [30].

**Figure 9:**
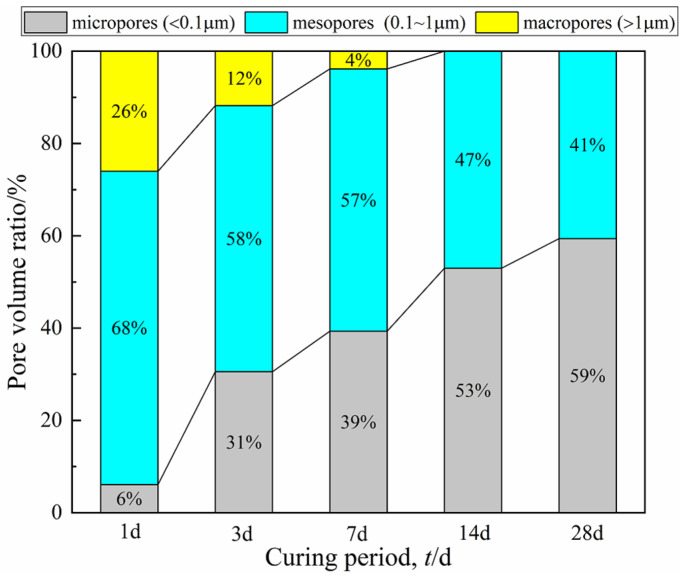
The pore structure evolution of the specimen with 15% cement.

The pore structure evolution of the specimen with 15% cement was shown in Figure 9. It can be observed that within 7 days of curing, the mesopores ranging from 0.1~1 µm in all specimens constituted the majority of the pore space, accounting for more than 50% of the total pore volume. However, the proportion of mesopores and macropores gradually decreased with the curing period. Moreover, the percentage of micropores (with a diameter between 0.01 and 0.1 µm) increased significantly to more than 50% (53% and 59% for 14 and 28 days of curing, respectively), while the macropores (>1.0 µm) disappeared. This evolution of pore structure can be attributed to the cement stabilization effect, that is, gels such as calcium silicate hydrate (CSH) bound soil aggregates more tightly and expansive minerals such as ettringite (AFt) filled intergranular spaces, resulting in the transformation of large pores into smaller ones.

**Figure 10:**
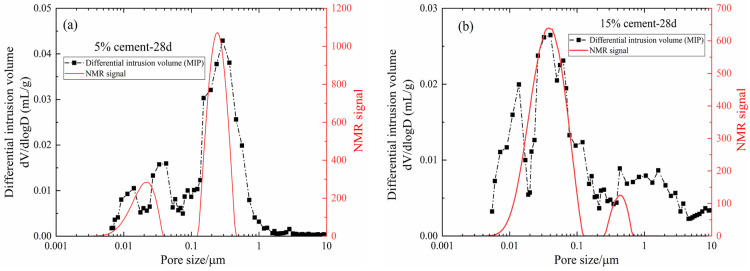
Comparison of MIP and NMR experimental results of specimens cured for 28 days: (**a**) 5% cement content, (**b**) 15% cement content.

MIP is a mature method to determine the pore structure of porous materials. In this study, the MIP results (differential intrusion volume curves) of specimens with 5% and 15% cement after 28 days of curing were compared with the pore size distribution determined by NMR, which is shown in Figure 10. Furthermore, the following conclusions can be drawn: the characteristics of the pore size distribution curves determined by the MIP and NMR were highly consistent, and the optimal pore sizes were also very close to each other. However, the pore sizes measured by MIP were generally slightly larger than those determined by NMR, especially in the range of pore diameters greater than 0.5 µm, and the pore size distribution curve determined by MIP was not smooth. The reason may be that in the process of the mercury injection test, the larger mercury pressure destroyed the skeleton structure of CDS and caused internal crack propagation. In general, this proved the reliability and accuracy of the NMR technique as a rapid and non-destructive method for detecting the pore structure of geotechnical media.

### 3.2. Geomechanical Properties (Strength and Permeability)

#### 3.2.1. Stress–Strain Relationship and Strength Development

**Figure 11:**
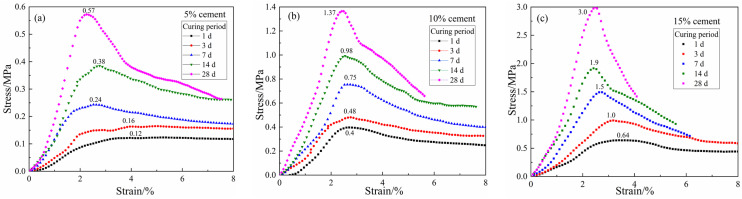
Stress–strain curves of CDS: (**a**) 5% cement content, (**b**) 10% cement content, (**c**) 15% cement content.

Figure 11 shows the stress–strain curves of CDS specimens at 1, 3, 7, 14, and 28 days of curing. The CDS specimens cured for one day all showed significant ductility, regardless of the cement content. When the curing period exceeded seven days, the cured specimens became more brittle with obvious peak strength. The increase in the brittleness and strength of CDS was caused by the cementation structure of soil aggregates. That is, the more hydrates (characterized by *m*_cw_, as shown in Figure 5) produced by cement hydration resulted in a denser and firmer microstructure of the solidified matrix, and the reduced pore water also exerted a weaker lubrication effect on the specimens under external compression. It can be seen that *m*_cw_ may be a suitable parameter to reflect the structure formed by the interaction between hydrates and soil aggregates in CDS. Therefore, it is necessary to further explore the quantitative relationship between the strength and water distribution of CDS.

**Figure 12:**
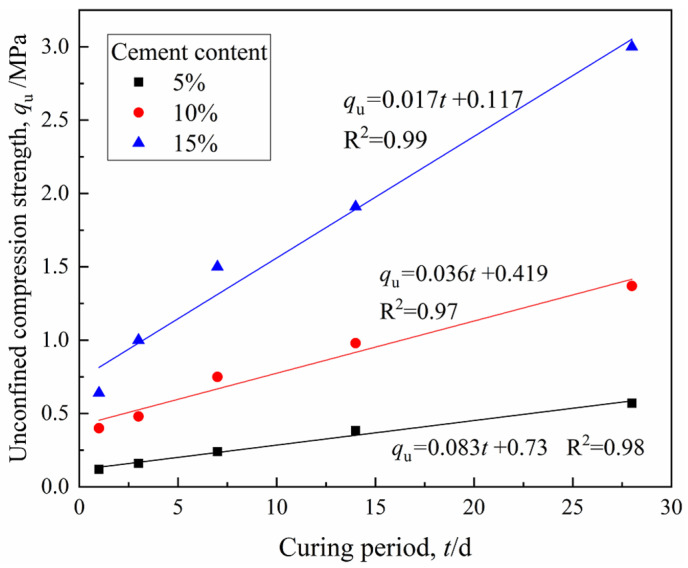
Unconfined compressive strength development versus curing period.

**Figure 13:**
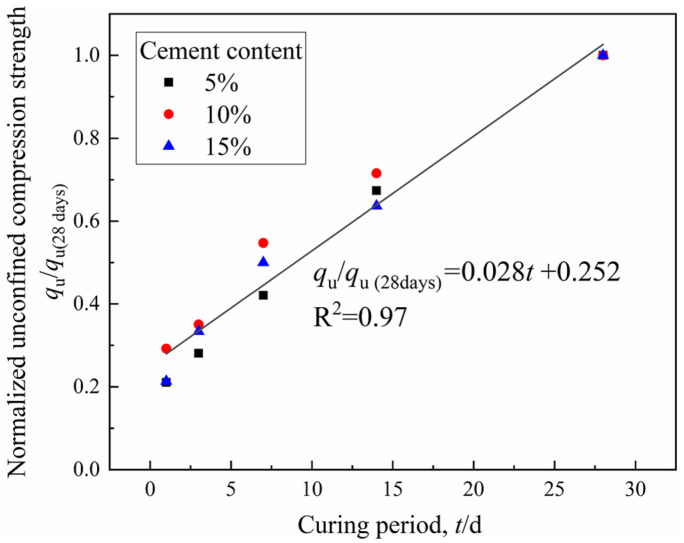
Variation of normalized strength with curing period.

The strength development with curing time is shown in Figure 12. It is noted that there is a linear relationship between the UCS (MPa) and curing period (days). The increase of strength with the curing period was due to the fact that hydration is a time-dependent process. On the other hand, the difference in the strength of 1-day specimens and 28-day specimens tended to increase for CDS with a higher cement content. Furthermore, the normalized strength (labeled as *q*_u_/*q*_u−28d_) can be obtained by dividing the strength of a particular curing time by the 28-day strength. In addition, the relationship between the normalized strength and curing period can be expressed by the following formula for specimens with different cement contents in this study (vide Figure 13).

(6) $$q_{u}/q_{u-28d}=0.252+0.028\times t$$

where *q*_u_ is the strength to be estimated at *t* days of curing, *q*_u−28d_ is the 28-day curing strength, and *t* is the curing time (days). The fitting results are of very good quality as indicated by the high coefficient of determination (R^2^ > 0.96). The inevitable scatter of the normalized *q*_u_ data may be due to the variability of UCT specimens at different curing ages.

#### 3.2.2. Hydraulic Conductivity

**Figure 14:**
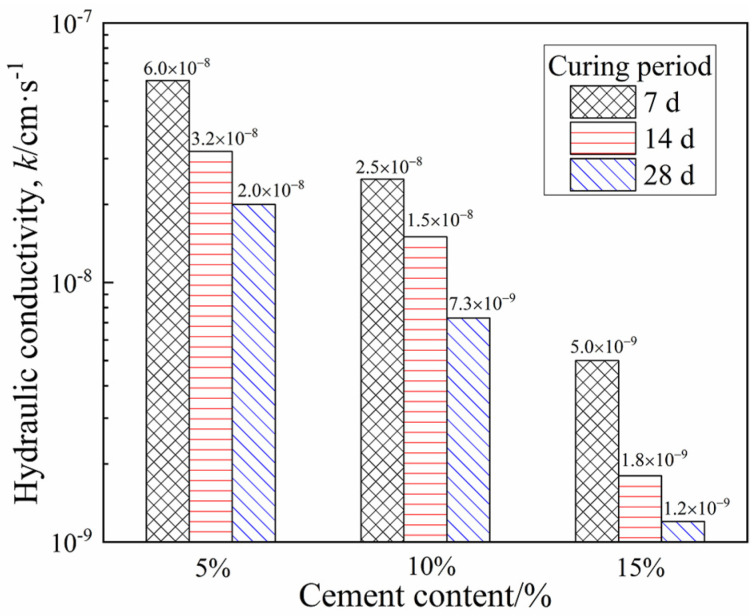
The hydraulic conductivity of CDS.

Figure 14 presents the hydraulic conductivity change of CDS and shows that prolonging the curing period reduced the permeability. Furthermore, the decreased rate of hydraulic conductivity *k* from 7 days to 28 days (i.e., $(k_{7d}-k_{28d})/k_{7d}\times 100\%$) tended to increase for CDS with higher cement content, indicating that the effect of the curing period was related to the cement content. It is worth mentioning that the increase in cement content also reduced the hydraulic conductivity, which was consistent with the experimental results of the cement-stabilized marine clay with metakaolin by Deng [29]. This can be attributed to the decrease of porosity and average pore size with the increase of hydrates, which led to increased resistance to water flow through the CDS [31].

### 3.3. Quantitative Relationship between Macro-Geomechanical Properties and Microstructure

#### 3.3.1. Relationship between Pore Structure Characteristics and Hydraulic Conductivity

**Figure 15:**
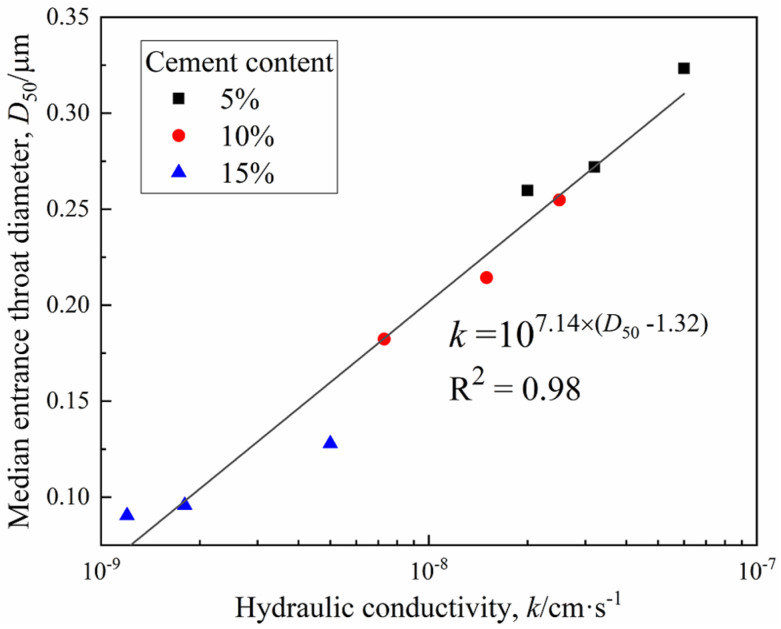
Relationship between *k* and *D*_50_.

According to Poiseuille’s theory of laminar flow in porous media, the permeability of water depends on the pore size of the materials [32]. Existing studies have shown the effectiveness of *D*_50_ as a representative parameter to characterize pore size distribution. Through detailed analysis of the above data, the permeability of CDS is intrinsically related to its pore size characteristics. Consequently, the relationship between hydraulic conductivity *k* and *D*_50_ in this study is presented in Figure 15, which further confirmed that hydraulic conductivity is the macro-representation reflection of micropore structures [33,34,35].

#### 3.3.2. Relationship between Strength and Water Distribution

**Figure 16:**
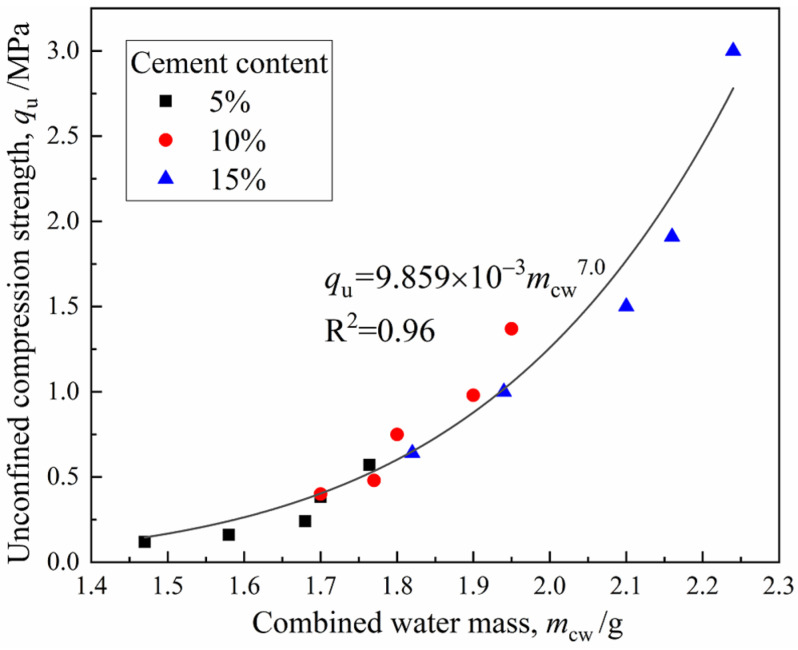
Relationship between *q*_u_ and *m*_cw_.

The correlation of the unconfined compressive strength *q*_u_ with combined water *m*_cw_ can be obtained by fitting the data from Figure 5 and Figure 12, as shown in Figure 16. The *q*_u_ of CDS can be well normalized by the parameter *m*_cw_ irrespective of the curing period and cement content. This observation is highly consistent with the results presented in the reference from Zhu et al. [20], in which three kinds of sediments were solidified with cement and the exponential function relationship between *q*_u_ and *m*_cw_ was established by a centrifugal method. It can be observed that *q*_u_ increased exponentially with *m*_cw_, which represented the number of hydrates and was attributed to the fact that, during the later curing period, the three-dimensional skeleton of the CDS matrix had been formed and the cementation and filling effect of the regenerated hydrates were more significant. In addition, most of the existing empirical models for strength development accounted for cement content and curing age generally at a certain initial water content. However, in this study, the parameter *m*_cw_ can comprehensively characterize the influence of the two factors mentioned above on the strength development of CDS from the view of a stabilization mechanism.

### 3.4. SEM Characterization

**Figure 17:**
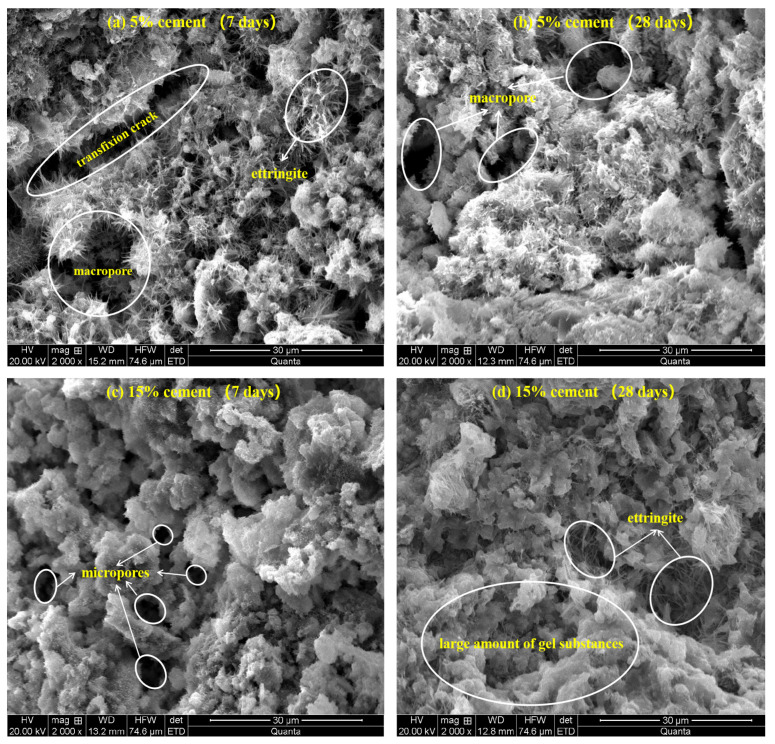
The micromorphology of stabilized specimens: (**a**) 5% cement-7 days, (**b**) 5% cement-28 days, (**c**) 15% cement-7 days, (**d**) 15% cement-28 days.

The micromorphology of the stabilized specimens with 5% and 15% cement at 7 and 28 days of curing is shown in Figure 17a–d. For the specimens with 5% cement cured for seven days (Figure 17a), a certain amount of acicular ettringite (AFt) can be observed, while a small amount of gel hydrate resulted in an obvious loose skeleton of solidified matrix with macropores and transfixion cracks, thus showing lower strength and higher hydraulic conductivity. After curing for 28 days (Figure 17b), although the fabric became relatively denser, the intergranular pores larger than 5 microns still existed. For the specimens with 15% cement cured for 7 days (Figure 17c), the soil particles were encapsulated and cemented by a large number of gel substances such as C-S-H, and the large pores disappeared. Furthermore, ettringite appeared again between the gap of aggregates within specimens cured for 28 days, and the coupling effect of ettringite filling and gel cementation led to an increase in the proportion of micropores and non-transfixable pores, which is presented in Figure 17d. Consequently, this denser and stronger structure of the CDS matrix showed superior strength and poor permeability (Figure 12 and Figure 14). Therefore, the microstructure characteristics measured by SEM intuitively reveal the evolution mechanism of strength and permeability, which were also highly consistent with NMR results. It should be pointed out that the pore diameter shown by SEM was slightly larger than that measured by the NMR technique because the pore size was expanded by the frost–heave force generated during the freeze-drying process.

## 4. Discussion

The microscopic testing methods, such as SEM, MIP, XRD, and TGA (thermogravimetric analysis), mainly explore the cement stabilization mechanism from the perspectives of micromorphology, pore structure, and mineral composition. However, the deficiencies of these methods are as follows: (1) The integrity and initial structure of the original specimen are inevitably damaged by the complex pretreatment, such as the freeze-drying process in the SEM and MIP tests; (2) a small amount of test sample taken locally (tens of micrograms or a few grams) may not be adequately representated, such as the powder sample used in XRD and TGA experiments; (3) the hydration process of the test specimen is forced to stop, making it difficult to carry out continuous tests on the same specimen throughout the curing period; and (4) the number of hydrates generated inside a specimen cannot be measured accurately, hence the essential relationship between the geomechanical behaviors and hydration reaction cannot be constructed quantitatively. In contrast, the NMR technology is used to test a full-size initial specimen directly, without any pretreatment that may cause microdamage inside the specimen. Furthermore, the NMR test can be carried out for the whole curing period of the same specimen, and each measurement only lasts for several minutes. The hydration progress can be accurately tracked by monitoring the conversion of pore water and combined water, and the pore structure characteristics can be deduced from the *T*_2_ distribution curve. Therefore, compared with the above traditional microscopic testing methods, the NMR technique is a non-destructive, continuous, straightforward, and simple method to study the stabilization mechanism of CDS, while providing convincing results. 

## 5. Conclusions

(1) The total peak area and the *T*_2_ at peak gradually decreased with an increasing curing period and cement content, indicating that more liquid pore water inside the CDS specimen transformed into combined water with cement hydration. The amount of combined water, *m*_cw_, which essentially characterized the hydration process of cement, presented a linear relationship with log (*t*).
(2) The cementation and filling action of hydrates resulted in the transformation of large pores into smaller ones, then the proportion of mesopores and macropores gradually decreased with the curing period. In addition, the characteristics of the pore size distribution curves determined by the MIP and NMR were highly consistent. Given that there was an essential relationship between permeability and pore size distribution, the functional correlations of hydraulic conductivity *k* and micropore parameters *D*_50_ were established.
(3) With the increase in the curing period, more hydrates contributed to the denser and firmer microstructure within the solidified matrix, and the stress–strain characteristic of CDS specimens changed from ductility to brittleness. Furthermore, the relationship between the normalized strength *q*_u_/*q*_u−28d_ and curing period *t* can be characterized by a linear function.
(4) The parameter *m*_cw_ can comprehensively characterize the influence of the curing period and cement content on the strength development of CDS from the view of a stabilization mechanism and *q*_u_ increased exponentially with *m*_cw_. The microstructure characteristics measured by SEM revealed the evolution mechanism of strength and permeability more intuitively.
(5) In the actual project of filling subgrade with CDS, increasing cement content can improve the strength and reduce hydraulic conductivity in a short term, which is helpful to promote the progress of some special projects with tight construction periods. In general, the curing period should be extended to at least 28 days, which is conducive to the full hydration and stabilization effect of cement.

## Figures and Tables

**Table 1:** Physical characteristics of dredged sediment.

Parameters	Values
Specific gravity	2.71
Liquid limit, %	50.0
Plastic limit, %	25.0
Plastic index (*I_P_*)	25.2
Clay fraction (d < 0.005 mm), %	21.0
Silt fraction (0.005 mm < d < 0.075 mm), %	64.0
Sand fraction (d > 0.075 mm), %	15.0

**Table 2:** Properties of cement utilized for this experiment.

Physical Property	Value	Chemical Composition (%)	Value	Mineral Composition	Value
Ignition loss, %	3.76	Silica, SiO_2_	21.3	C_3_S, %	56.54
Specific gravity	3.13	Calcium oxide, CaO	64.8	C_2_S, %	22.56
Fineness, m^2^/kg	354	Alumina, Al_2_O_3_	5.2	C_3_A, %	8.32
Initial setting time, min	208	Ferric oxide, Fe_2_O_3_	3.3	C_4_AF, %	10.32
Final setting time, min	258	Magnesium oxide, MgO	2.47		
UCS ^a^ in 3 d, MPa	30.3	Chloride, Cl^−^	0.021		
UCS in 28 d, MPa	43.2	Sulfur oxide, SO_3_	2.83		
		Sodium oxide, Na_2_O	0.08		

Note: ^a^ means unconfined compression strength.

**Table 3:** Mix design and testing program.

Test Items	Curing Period (Days)	Cement Content (%)
NMR	1, 3, 7, 14, 28	5%, 10%, 15%
UCS	1, 3, 7, 14, 28	5%, 10%, 15%
HCT	7, 14, 28	5%, 10%, 15%
SEM	7, 28	5%, 15%
MIP	28	5%, 15%

## Data Availability

The data that support the findings of this study are available on request from the corresponding authors.
